# A 2:1 formulation of follitropin alfa and lutropin alfa in routine clinical practice: a large, multicentre, observational study

**DOI:** 10.3109/09513590.2010.511014

**Published:** 2010-09-17

**Authors:** Klaus Bühler, Olaf Naether

**Affiliations:** 1 Centre for Reproductive Medicine and Gynaecological Endocrinology, Langenhagen, Germany; 2 Fertility Center Hamburg, Hamburg, Germany

**Keywords:** Assisted reproductive technology, follitropin alfa, hypogonadotrophic hypogonadism, lutropin alfa, ovarian stimulation

## Abstract

**Background:**

A 2:1 (150 IU:75 IU) follitropin alfa:lutropin alfa formulation has been developed. A 3-year post-marketing surveillance study is ongoing in Germany to explore the use of this formulation in routine clinical practice.

**Materials and methods:**

An 11-month interim analysis of data from assisted reproductive technology (ART) cycles only is described.

**Results:**

Data were available from 857 patients undergoing 919 cycles of ART at 19 centres. Most patients (58.7%) were aged ≥35 years, and many (41.3%) were undergoing their first ART cycle. Main reasons cited by physicians for prescribing this formulation were poor response in a previous treatment cycle (*n* = 303) and low basal luteinizing hormone (LH) level (*n* = 107). Mean (standard deviation) duration of ovarian stimulation was 10.8 (2.6) days. In 90.7% of cycles, the 2:1 formulation was administered throughout the stimulation period. Most frequent LH daily dose was 75 IU. Embryo transfer was conducted in 741 cycles; clinical pregnancy rate per transfer was 27.5%. Three cases of ovarian hyperstimulation syndrome developed in three patients (3/741 [0.4%] cycles); one required hospitalization. No other major safety events were reported.

**Conclusion:**

This interim analysis shows that use of the 2:1 formulation for ovarian stimulation during routine ART procedures is effective in achieving clinical pregnancies and is associated with a positive safety profile.

## Introduction

Follicle stimulating hormone (FSH) and luteinizing hormone (LH) are essential for the development of ovarian follicles and subsequent stages of maturation and ovulation [1]. Severely reduced hypothalamic or pituitary activity results in LH and FSH deficiency (WHO Group I anovulatory infertility; hypogonadotrophic hypogonadism [HH]). Administration of exogenous FSH in combination with LH has proven to be effective in promoting follicular maturation, ovulation and pregnancy in women with HH [2-5]. Both FSH and LH are commercially available as urinary-derived or recombinant hormones. Unlike urinary-derived hormones, recombinant formulations provide a pure and consistent source of gonadotrophins [6].

A 2:1 formulation of recombinant human (r-h)FSH and r-hLH (in a fixed-dose combination of 150 IU r-hFSH [follitropin alfa]:75 IU r-hLH [lutropin alfa]) has been developed. In the combined formulation, there are no pharmacokinetic or pharmacodynamic interactions between the constituent gonadotrophins, and their bioactivity is unaltered [7-10], thus, allowing the administration of both hormones in a single injection. By avoiding the need for two separate injections or mixing of gonadotrophins prior to injection, the 2:1 formulation of follitropin alfa and lutropin alfa offers potential benefits for patient convenience.

Although the majority of normogonadotrophic women have adequate endogenous LH levels, certain patient subgroups may benefit from LH supplementation during assisted reproductive technology (ART) [11,12]. Accordingly, physicians have attempted to exploit the potential benefits of the 2:1 formulation of follitropin alfa and lutropin alfa in a variety of patient populations.

The 2:1 formulation of follitropin alfa and lutropin alfa has been available in Germany since October 2007 for the stimulation of follicular development in women with severe FSH and LH deficiency (defined in clinical trials by an endogenous serum LH level of < 1.2 IU/1) [3,13]. A 3-year post-marketing surveillance study is in progress in Germany to explore the use of the 2:1 formulation of follitropin alfa and lutropin alfa in routine clinical practice. The study aims to define the characteristics of patients with LH deficiency in daily practice and learn about the ovarian stimulation protocols that such patients undergo. Here, we present an interim analysis of data on ART cycles collected after 11 months.

## Methods

### Study design

An observational, post-marketing surveillance study was initiated across *in vitro* fertilisation (IVF) centres in Germany. The aim of the study is to collect data on 5000 patients at 49 centres between January 2008 and December 2010. An interim analysis was performed on data collected between January and November 2008 from ART cycles only.

### Assessments

Routine clinical data for patients undergoing cycles of conventional IVF or intracytoplasmic sperm injection (ICSI) were entered prospectively into an electronic database system (RecDate [14]). Data from more than one cycle per patient were permitted.

A questionnaire prior to initiation of treatment with the 2:1 formulation of follitropin alfa and lutropin alfa (Merck Serono S.A. - Geneva, Switzerland, an affiliate of Merck KGaA, Darmstadt, Germany) was completed by the clinician of each individual patient. One or more of the following reasons for prescribing the therapy could be selected: low serum LH level, low serum oestrogen level, thin endometrium, amenorrhoea, or ‘other’. If ‘other’ was selected, the clinician was prompted to specify the reason(s) using a free text box.

### Statistical analysis

Endpoints included the prescribed dose and duration of treatment with the 2:1 formulation of follitropin alfa and lutropin alfa.

In accordance with the design and objective of the study, statistical evaluation focussed on a summary and detailed report of the data obtained by means of descriptive statistics (means, standard deviations [SD], frequencies, percentages). No statistical hypotheses were pre-specified, and no statistical tests were performed.

## Results

### Patient characteristics

At the time of this analysis, data were available from 19 German IVF centres. In total, 857 patients had undergone 919 cycles of ART using the 2:1 formulation of follitropin alfa and lutropin alfa. ICSI and IVF were performed in 73% and 23% of cycles, respectively.

Baseline demographic and clinical characteristics of the patients are shown in Table I. The majority of the study population (58.7%) were aged ≥35 years (range: 19.0–48.0 years). The number of previous ART cycles undertaken by the 857 patients is shown in Table II; this was the first ART cycle for just over 40% of patients.

**Table I:** Baseline patient characteristics.

Variable	Number of patients analysed	Mean (SD)
Age, years	838	34.8 (4.5)
BMI, kg/m^2^	848	23.1 (3.7)
Serum LH level, IU/1	743	5.5 (8.5)
Serum FSH level, IU/1	421	11.3 (13.0)
Serum oestradiol level, pg/ml	721	37.4 (36.0)
AFC (number of follicles < 11 mm)	279	6.7 (3.7)

AFC, antral follicle count; BMI, body mass index; FSH, follicle-stimulating hormone; LH, luteinizing hormone; SD, standard deviation.

**Table II:** Assisted reproductive technology cycle history.

Number of previous ART cycles	Patients *n* (%)
0	354 (41.3)
1	200 (23.3)
2	143 (16.7)
≥3	160 (18.7)

Total number of patients analysed = 857.

### Reasons for prescribing the 2:1 formulation of follitropin alfa and lutropin alfa

**Figure 1:**
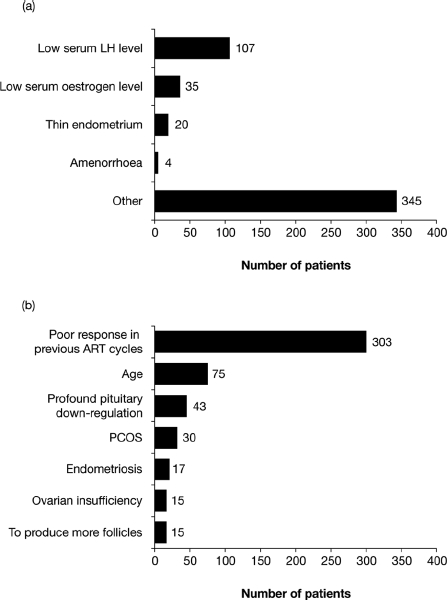
Most common reasons cited by clinicians for prescribing the 2:1 formulation of follitropin alfa and lutropin alfa. (a) The number of patients who were prescribed the 2:1 formulation of follitropin alfa and lutropin alfa for one (or more) of the pre-specified reasons, (b) The number of patients who were prescribed the 2:1 formulation of follitropin alfa and lutropin alfa for ‘other’ reasons (the seven reasons most frequently cited by clinicians are shown). ART, assisted reproductive technology; LH, luteinizing hormone; PCOS, polycystic ovarian syndrome.

The main reasons cited by clinicians for prescribing the 2:1 formulation of follitropin alfa and lutropin alfa are shown in Figure 1. Low serum LH level was cited as the reason for prescribing the 2:1 formulation of follitropin alfa and lutropin alfa in 107 patients (Figure 1a). The most common ‘other’ reason cited for prescribing the 2:1 formulation of follitropin alfa and lutropin alfa was a previous poor response in ART cycles (303 patients; Figure 1b).

### Interventions

The proportions of patients who received various drug treatments are shown in Table III. Approximately twice as many cycles involved the use of gonadotrophin-releasing hormone (GnRH) agonists as antagonists for pituitary suppression. Most (95.5%) of the GnRH-agonist cycles were performed using a long protocol.

**Table III:** Concomitant treatments received during assisted reproductive technology cycles.

Treatment	Number of cycles analysed	Cycles *n* (%)
Contraceptive pill (cycle programming)	885	283 (32.0)
GnRH antagonist	919	288 (31.3)
GnRH agonist	919	618 (67.2)
Long protocol	618	590 (95.5)
Intranasal administration	618	431 (69.7)

GnRH, gonadotrophin-releasing hormone.

The mean (SD) duration of ovarian stimulation was 10.8 (2.6) days. The mean (SD) total dose of r-hFSH and r-hLH received per cycle was 2644.4 (1178.3) IU and 1185.5 (572.1) IU, respectively.

Of the 919 cycles documented, information relating to the dose of the 2:1 formulation of follitropin alfa and lutropin alfa was available for 654 cycles. In 593 (90.7% of cycles analysed) cycles, the 2:1 formulation of follitropin alfa and lutropin alfa was administered throughout ovarian stimulation. Of these, a consistent daily dose of 75 IU LH was received in 351 (59.2%) cycles, a consistent daily dose of 150 IU LH was received in 171 (28.8%) cycles and a consistent daily dose of 225 IU LH was received in 23 (3.9%) of cycles. A dose adjustment of the 2:1 formulation of follitropin alfa and lutropin alfa was made during the stimulation period in the remaining cycles: the dose was simply altered in 41 (6.9%) cycles, whereas in 7 (1.2%) cycles additional r-hFSH was given and consequently the FSH:LH ratio was greater than 2:1.

### Effectiveness and safety data

Of the 919 cycles documented, human chorionic gonadotrophin was administered as scheduled in 877 (95.4%) cycles, oocyte pick-up was performed in 858 (93.4%) cycles and insemination via IVF or ICSI was achieved in 831 (90.4%) cycles. Fresh embryo transfer was conducted in 741 (89.2%) of these cycles.

The mean (SD) number of oocytes retrieved per patient was 8.6 (5.6), of which a mean number of 6.8 (79.5%) were mature oocytes. A total of 62.5% of inseminated/injected oocytes were fertilized and developed to the two pronuclear (2PN) stage of development (mean [SD] of 4.1 [3.1] 2PN oocytes per patient).

The number of embryos transferred is shown in Table IV. The mean (SD) number of embryos transferred per cycle was 2.02 (0.61). The implantation rate per embryo transferred was 15.9% (238/1499). Clinical pregnancy was achieved after approximately one quarter of embryo transfer procedures (204/741, 27.5%). Daily doses of 150 IU r-hFSH:75 IU r-hLH and 300 IU r-hFSH:150 IU r-hLH resulted in clinical pregnancy rates per embryo transfer of 32.1% and 16.3%, respectively.

**Table IV:** Number of fresh embryos transferred following ovarian stimulation using the 2:1 formulation of follitropin alfa and lutropin alfa.

Outcome	Number of cycles analysed	Cycles *n* (%)
Number of embryos transferred:		
1	741	129 (17.4)
2	741	466 (62.9)
3	741	146 (19.7)

A total of 204 pregnancies were recorded, and information was available on the outcome of 106 pregnancies. In total, 78 babies were born from 60 pregnancies: 43 singleton and 17 multiple deliveries (16 pairs of twins and 1 set of triplets). Five babies had congenital abnormalities (three singleton and two multiple births) and eight required admission to neonatal intensive care (two singletons and six from multiple births). There were 41 spontaneous miscarriages and five ectopic pregnancies.

Three cases of ovarian hyperstimulation syndrome (OHSS) were reported in three patients (3/741 [0.4%] cycles that resulted in embryo transfer). Two of the cases of OHSS were considered severe (Grade III OHSS) but did not require hospitalization. One case of OHSS (that occurred in a cycle in which pregnancy was achieved) required hospitalization. The 34-year-old patient, who had hyperprolactinaemia, underwent ovarian stimulation for 9 days with a total dose of 2550:1275 IU of r-hFSH:r-hLH. She subsequently made a full recovery from OHSS. No other safety events were reported in this observational study.

## Discussion

There is little available data on the use of LH supplementation in ART clinical practice with respect to the most suitable treatment protocol, the optimal timing of LH administration, or the most appropriate dose of LH. This 3-year post-marketing surveillance study will explore the potential role of LH, and evaluate use of the 2:1 formulation of follitropin alfa and lutropin alfa, in routine clinical practice.

A total of 857 patients undergoing 919 cycles of ART were included in this interim analysis. Of these, a low serum LH level at baseline was the reason cited for prescribing the 2:1 formulation of follitropin alfa and lutropin alfa for 107 patients. However, the most commonly cited reason was a previous poor response to ovarian stimulation (303 patients).

GnRH agonists were used approximately twice as frequently as antagonists. The vast majority (95.5%) of the GnRH-agonist cycles were performed using a long protocol. The 2:1 formulation of follitropin alfa and lutropin alfa was given throughout the whole ovarian stimulation period in most cases (90.7% of cycles), and the most commonly used daily dose of r-hLH (in over half of the cycles) was 75 IU.

The use of the 2:1 formulation of follitropin alfa and lutropin alfa for ovarian stimulation during routine ART procedures was effective in achieving clinical pregnancies. The overall clinical pregnancy rate observed in this study was 27.5%. This is comparable with, albeit slightly lower than, data from a recently published analysis of results generated from European registers, in which the clinical pregnancy rates for IVF and ICSI were 30.3% and 30.9%, respectively [15]. The slightly lower pregnancy rate reported in the current observational study may be a reflection of the enrolled study population; almost 60% of the patients were aged at least 35 years, and one-third had previously experienced a poor response to ovarian stimulation. Indeed, a pregnancy rate of 24.4% was recently reported among women aged at least 35 years who underwent IVF or ICSI in Germany [16].

The clinical pregnancy rate in this study was highest among those who received a daily dose of 150 IU r-hFSH:75 IU r-hLH (32.1% vs. 16.3% for those receiving 300 IU r-hFSH:150 IU r-hLH per day). However, it should also be noted that women who were prescribed 300 IU r-hFSH:150 IU r-hLH per day were likely to have been expected to have a poor response to ovarian stimulation.

In accordance with a previous report [10], the 2:1 formulation of follitropin alfa and lutropin alfa was found to have a favourable safety profile. Only three patients developed OHSS (3/741, 0.4% of cycles resulting in embryo transfer), of which only one required hospitalization. Among the 60 pregnancies, there were 17 multiple pregnancies, including one triplet pregnancy.

The nature of this observational, post-marketing surveillance study must be acknowledged. Post-marketing surveillance studies rely on accurate reporting of events. As such, some data sets in this study are incomplete (and the number of patients with evaluable data was different for each outcome). Furthermore, due to the nature of the questionnaire, there may be potential sources of variability among responses submitted by different physicians. For example, various definitions of a ‘poor response to stimulation’ may have been used by individual physicians. Although this was not a randomized, comparative study, the RecDate database collects information from approximately 85% of all German IVF centres. Thus, the findings of this observational study are considered to be reflective of normal clinical ART practice in Germany, and offer a valuable insight into the use of the 2:1 formulation of follitropin alfa and lutropin alfa.

In conclusion, the interim results of this large observational study of the use of the 2:1 formulation of follitropin alfa and lutropin alfa for ovarian stimulation (in 857 patients and 919 cycles of ART) show that it is associated with a positive safety and effectiveness profile in routine clinical practice. The most commonly cited reasons for prescribing the 2:1 formulation of follitropin alfa and lutropin alfa were a low serum LH level and a previous poor response to ovarian stimulation. Thus, the 2:1 formulation of follitropin alfa and lutropin alfa may be beneficial for women in either of these subgroups. The study is currently ongoing, with the final data expected to be available in 2011.

## References

[1] Chappel SC, Howles C (1991). Reevaluation of the roles of luteinizing hormone and follicle-stimulating hormone in the ovulatory process. Hum Reprod.

[2] Burgues S (2001). The effectiveness and safety of recombinant human LH to support follicular development induced by recombinant human FSH in WHO group I anovulation: evidence from a multicentre study in Spain. Hum Reprod.

[3] Kaufmann R, Dunn R, Vaughn T, Hughes G, O'Brien F, Hemsey G, Thomson B, O'Dea LS (2007). Recombinant human luteinizing hormone, lutropin alfa, for the induction of follicular development and pregnancy in profoundly gonadotrophin-deficient women. Clin Endocrinol (Oxf).

[4] Shoham Z, Smith H, Yeko T, O'Brien F, Hemsey G, O'Dea L (2008). Recombinant LH (lutropin alfa) for the treatment of hypogonadotrophic women with profound LH deficiency: a randomized, double-blind, placebo-controlled, proof-of-efficacy study. Clin Endocrinol (Oxf).

[5] The European Recombinant Human LH Study Group (1998). Recombinant human luteinizing hormone (LH) to support recombinant human follicle-stimulating hormone (FSH)-induced follicular development in LH- and FSH-deficient anovulatory women: a dose-finding study. J Clin Endocrinol Metab.

[6] Bassett R, Lispi M, Ceccarelli D, Grimaldi L, Mancinelli M, Martelli F, Dorsselaer A (2009). Analytical identification of additional impurities in urinary-derived gonadotrophins. Reprod Biomed Online.

[7] Agostinetto R (2009). Administration of follitropin alfa and lutropin alfa combined in a single injection: a feasibility assessment. Reprod Biol Endocrinol.

[8] Alper M, Meyer R, Dekkers C, Ezcurra D, Schertz J, Kelly E (2008). Assessment of the biopotency of follitropin alfa and lutropin alfa combined in one injection: a comparative trial in Sprague-Dawley rats. Reprod Biol Endocrinol.

[9] le Cotonnec JY, Loumaye E, Porchet HC, Beltrami V, Munafo A (1998). Pharmacokinetic and pharmacodynamic interactions between recombinant human luteinizing hormone and recombinant human follicle-stimulating hormone. Fertil Steril.

[10] Picard M, Rossier C, Papasouliotis O, Lugan I (2008). Bioequivalence of recombinant human FSH and recombinant human LH in a fixed 2:1 combination: two phase I, randomised, crossover studies. Curr Med Res Opin.

[11] Alviggi C, Mollo A, Clarizia R, De Placido G (2006). Exploiting LH in ovarian stimulation. Reprod Biomed Online.

[12] Humaidan P, Bungum M, Bungum L, Yding Andersen C (2004). Effects of recombinant LH supplementation in women undergoing assisted reproduction with GnRH agonist down-regulation and stimulation with recombinant FSH: an opening study. Reprod Biomed Online.

[13] O'Dea L, O'Brien F, Currie K, Hemsey G (2008). Follicular development induced by recombinant luteinizing hormone (LH) and follicle-stimulating hormone (FSH) in anovulatory women with LH and FSH deficiency: evidence of a threshold effect. Curr Med Res Opin.

[14] Pak SJ, Warlich J, van Rooij TN (2001). [RecDate - an IT-solution for the documentation and quality management of reproductive medicine]. Zentralbl Gynakol.

[15] Nyboe Andersen A, Goossens V, Bhattacharya S, Ferraretti AP, Kupka MS, de Mouzon J, Nygren KG (2009). Assisted reproductive technology and intrauterine inseminations in Europe, 2005: results generated from European registers by ESHRE: ESHRE. The European IVF Monitoring Programme (EIM), for the European Society of Human Reproduction and Embryology (ESHRE). Hum Reprod.

[16] German IVF Registry http://www.meb.uni-bonn.de/frauen/DIR_downloads/dirjahrbuch2007.pdf.

